# Ambient fine-particulate air pollution associates with short sleep duration among 2,082 community-dwelling older adults: findings from a large-scale questionnaire survey

**DOI:** 10.3389/fpubh.2026.1673763

**Published:** 2026-03-18

**Authors:** Ning Liang, Yitong Zhou, Wei Ran, Ruixue Yuan

**Affiliations:** 1 Department of Anesthesiology, The First Affiliated Hospital of Chongqing Medical University, Chongqing, China; 2 Department of Geriatrics, The Affiliated Hospital of Sichuan North Medical College, Nanchong, China

**Keywords:** ambient PM2.5, community-dwelling older adults, environmental co-exposures, particulate air pollution, short sleep duration, sleep disparities

## Abstract

**Objective:**

Short sleep duration is widespread in China’s aging population, yet the contribution of ambient fine-particulate matter (PM₂.₅) remains uncertain. We investigated whether chronic PM₂.₅ exposure associates with habitual short sleep duration.

**Methods:**

Within a hospital-based outreach program we surveyed 2082 community-dwelling adults aged ≥60 years in Nanchong and Chongqing (January–December 2024). Annual PM₂.₅ (1 km^2^ resolution) was estimated via a validated satellite–ground-fusion model; sleep duration was self-reported using the Chinese version of the Pittsburgh Sleep Quality Index, which has been culturally adapted and validated against actigraphy (*ρ* = 0.62). Short sleep duration was predefined as <6 h night^−1^. Mixed-effects logistic regression, adjusted for socioeconomic, lifestyle, cardiometabolic and environmental covariates, quantified associations per 10 μg m^−3^ increment and across exposure strata (<35, 35–50, and >50 μg m^−3^). Restricted cubic splines explored non-linearity, while population-attributable fractions and C-index shifts appraised public-health impact and predictive gain.

**Results:**

Mean PM₂.₅ was 44.1 ± 12.5 μg m^−3^; 27.3% of participants reported short sleep duration. Each 10 μg m^−3^ rise in PM₂.₅ increased short-sleep odds by 12% (adjusted OR 1.12, 95% CI 1.04–1.20). Compared with <35 μg m^−3^, exposure >50 μg m^−3^ conferred 51% higher odds (OR 1.51, 1.18–1.94). The relationship was monotonic and approximately log-linear. Achieving PM₂.₅ ≤ 35 μg m^−3^ could avert 24% of short-sleep cases; introducing PM₂.₅ improved discrimination from 0.55 to 0.57 (*Δ* 0.02).

**Conclusion:**

Chronic PM₂.₅ exposure is a modifiable, dose-dependent driver of short sleep duration in older Chinese adults. Air-quality control may yield meaningful sleep-health dividends.

## 1 Introduction

Short sleep duration has emerged as a pervasive, yet often under-recognized, geriatric health concern. In this study we focus specifically on short sleep duration, defined as self-reported nightly sleep of fewer than 6 h, and do not directly assess sleep fragmentation or other dimensions of sleep quality. Contemporary nationwide surveys indicate that nearly one in three Chinese adults aged ≥ 60 years habitually sleeps fewer than 6 h per night, a pattern independently linked to heightened risks of type 2 diabetes, cognitive decline, depressive symptomatology and all-cause mortality (1–4). With China’s population projected to rise to 430 million people aged 60 years or older by 2050, the societal and clinical ramifications of curtailed restorative sleep in late life are poised to intensify (5–7).

Alongside classic behavioral determinants—irregular bedtimes, sedentariness and caffeine or alcohol misuse—environmental exposures have garnered increasing attention as modifiable drivers of sleep disruption. Fine-particulate matter with an aerodynamic diameter ≤ 2.5 μm (PM₂.₅) is a ubiquitous pollutant that readily penetrates alveolar barriers, precipitates systemic inflammation and exerts downstream neuro-endocrine effects (8–11). Average annual PM₂.₅ concentrations across many Chinese prefectures still exceed the National Ambient Air Quality Standard Level 2 threshold (<35 μg m^−3^), rendering older residents chronically susceptible to pollutant burdens that outstrip those encountered in Europe or North America (12–15). Although the cardiopulmonary sequelae of PM₂.₅ are well characterized, its implications for sleep health remain comparatively underexplored.

Several interwoven biological pathways plausibly connect inhaled particulates to curtailed sleep duration. Translocated ultrafine particles and particle-induced cytokinaemia (e.g., interleukin-6, tumor-necrosis-factor-α) can breach the blood–brain barrier and perturb hypothalamic sleep-regulatory nuclei; oxidative stress dampens nocturnal melatonin synthesis and destabilizes peripheral circadian clocks; and pollutant-triggered autonomic imbalance, typified by sympathetic over-activity and reduced heart-rate variability, may delay sleep initiation while amplifying nocturnal arousals (16–18). Converging evidence from chamber experiments in rodents and observational cohorts in younger adults supports these mechanistic links, yet key knowledge gaps persist.

First, epidemiologic studies examining ambient PM₂.₅ and sleep have been limited by modest sample sizes (<1,200 participants), geographically restricted catchments, or reliance on coarse city-wide pollutant averages that obscure substantial intra-urban heterogeneity (19–22). Second, older adults—whose neuro-immunologic resilience is attenuated and whose sleep architecture is inherently vulnerable—have been markedly under-represented. Third, extant investigations seldom incorporate comprehensive adjustment for co-exposures such as ambient temperature, household solid-fuel combustion or noise annoyance, nor do they systematically evaluate potential effect modification by depressive symptoms or obesity, both highly prevalent in late life.

Against this backdrop, we leveraged a hospital-based community-outreach program in two south-western Chinese cities to assemble a large, well-characterized cohort of 2082 community-dwelling older adults. Ambient PM₂.₅ exposure was estimated with a high-resolution (1 km^2^) satellite–ground-fusion model, and sleep duration was ascertained via a validated Chinese-language questionnaire supported by actigraphic correlation. We posited three inter-related hypotheses: first, that habitual short sleep (<6 h night^−1^) would exhibit a dose-dependent rise across incremental PM₂.₅ strata; second, that each 10 μg m^−3^ increase in annual PM₂.₅ would translate into materially higher odds of short sleep after rigorous control for socioeconomic, lifestyle and environmental confounders; and third, that integrating PM₂.₅ exposure into parsimonious clinical models would confer incremental predictive discrimination for short sleep beyond traditional risk factors. By interrogating these hypotheses within a unified analytic framework, the present investigation aims to illuminate the sleep-health burden attributable to particulate pollution in an aging society and to inform multifaceted strategies that couple air-quality mitigation with geriatric sleep preservation.

## 2 Methods

### 2.1 Study design and participants

This cross-sectional investigation was embedded in a hospital-based community-outreach program that screened older adults for cardiopulmonary health in Nanchong (Sichuan Province) and Chongqing Municipality between 1 January 2024 and 31 December 2024. Consecutive residents aged ≥ 60 years living independently in the catchment areas of the Affiliated Hospital of North Sichuan Medical College and the First Affiliated Hospital of Chongqing Medical University were invited through primary-care rosters, neighborhood committees, and local media. Individuals were enrolled during face-to-face sessions at community clinics or hospital outpatient halls after verification of residential addresses. Exclusion criteria comprised physician-diagnosed obstructive sleep apnoea, night-shift employment within the preceding year, severe cognitive impairment (Mini-Mental State Examination < 18), or missing geolocation information. Among 2,279 screened volunteers, 197 were excluded (72 declined consent, 58 failed eligibility, 67 provided incomplete questionnaires), yielding an analytic sample of 2082 participants. All procedures conformed to the Declaration of Helsinki. The study was approved by the Institutional Review Board of the Affiliated Hospital of North Sichuan Medical College (NSMCAH6122-9014-85CZ). Written informed consent was obtained in Mandarin from every participant.

### 2.2 Exposure, outcome and covariate assessment

Ambient fine-particulate matter (PM₂.₅) exposure was estimated with a validated satellite–ground-fusion model that integrates 1 km^2^ aerosol optical depth retrievals, fixed-site monitoring data, land-use terms, and meteorological fields (temperature, humidity, planetary boundary-layer height). Daily PM₂.₅ surfaces for 2023 were generated in R (version 4.3.2) using a random-forest algorithm with 10-fold spatial cross-validation (mean *R*^2^ = 0.83). Each participant’s annual average PM₂.₅ concentration was calculated by overlaying residential coordinates (geocoded in QGIS 3.34) onto the exposure grid; values were treated as continuous (per 10 μg m^−3^ increment) and categorical [low <35, medium 35–50, high >50 μg m^−3^, reflecting Chinese National Ambient Air Quality Standard Level 2 (23)]. These annual averages were used as indicators of chronic exposure; short-term (e.g., daily or weekly) PM₂.₅ fluctuations were not modeled. For two-pollutant sensitivity analyses, annual average nitrogen dioxide (NO₂) at each residence was derived from companion prediction surfaces based on the national monitoring network and land-use covariates.

Sleep duration was self-reported via the Chinese version of the Pittsburgh Sleep Quality Index (“During the past month, how many hours of actual sleep did you get at night on average?”). The Chinese Pittsburgh Sleep Quality Index has demonstrated acceptable reliability and construct validity in community-dwelling adults and other Chinese populations (24, 25). Short sleep duration was predefined *a priori* as <6 h night^−1^, a threshold linked to adverse metabolic and neurocognitive outcomes in older Chinese cohorts (1–4). A subset of 280 individuals wore wrist actigraphs for 7 days; the questionnaire–actigraphy correlation (Spearman *ρ* = 0.62) supported construct validity in the present sample.

Covariates were captured through interviewer-administered questionnaires, physical examination, and electronic medical-record linkage: age, sex, educational attainment, household income, body-mass index, waist circumference, smoking (never/former/current), alcohol intake, tea consumption, physical-activity energy expenditure (International Physical Activity Questionnaire), hypertension, diabetes, depressive symptoms (Patient Health Questionnaire-9), chronic pain, indoor solid-fuel use, ambient noise annoyance (5-point Likert scale), season of interview, and mean ambient temperature in the month preceding the survey. Systolic and diastolic blood pressure were measured three times with an automated device; the average of the last two readings was recorded.

### 2.3 Statistical analysis

Analyses adhered to a pre-specified protocol written before data lock. Descriptive statistics are presented as mean ± SD, median (interquartile range), or percentage, and group differences were examined using *t*-tests, Mann–Whitney *U* tests, or *χ*^2^ tests as appropriate. Multivariable mixed-effects logistic regression quantified the association between PM₂.₅ and short sleep, with a random intercept for recruitment site to account for clustering. Model 1 adjusted for age and sex; Model 2 further adjusted for socioeconomic, lifestyle, and cardiometabolic factors; Model 3 incorporated environmental co-exposures (ambient temperature, noise, indoor solid-fuel use) to evaluate robustness. Odds ratios (ORs) with 95% confidence intervals (CIs) were expressed per 10 μg m^−3^ increase and across exposure categories, with linear trend assessed by modeling the median within each category as a continuous variable. Restricted cubic splines with four knots at the 5th, 35th, 65th, and 95th percentiles of the PM₂.₅ distribution were fitted on top of the fully adjusted covariate set (Model 3) using the rcs function in the rms package to explore potential non-linearity, and Wald *χ*^2^ tests were used to evaluate overall and non-linear components of the association. Effect modification by sex, obesity (BMI ≥ 28 kg m^−2^), and depressive symptoms was tested by introducing cross-product terms and comparing likelihood-ratio statistics. Multiple imputation by chained equations (20 datasets) handled missing covariates (<4% for any variable), and results were pooled using Rubin’s rules. Sensitivity analyses comprised (1) using an alternative short-sleep definition <5 h; (2) excluding participants reporting physician-diagnosed insomnia; (3) adding nitrogen dioxide (NO₂) to two-pollutant models to gauge co-pollutant confounding; (4) repeating models with actigraphy-derived sleep duration among the sub-sample. Population-attributable fractions were computed under the counterfactual scenario of PM₂.₅ ≤ 35 μg m^−3^. All tests were two-sided with *α* = 0.05. Analyses were performed in R 4.3.1.

## 3 Results

### 3.1 Participant characteristics

Among the 2082 community-dwelling older adults analyzed (55% from Nanchong, 45% from Chongqing), the mean age was 70.9 ± 6.4 years and 44.5% were women (Table 1). Overall, 568 individuals (27.3%) reported short sleep duration (<6 h night^−1^). Compared with their counterparts who slept ≥6 h, short-sleepers were slightly older (71.2 ± 6.4 vs. 70.8 ± 6.4 years), more frequently female (50.4% vs. 42.4%), and showed a higher prevalence of depressive symptoms (24.7% vs. 19.5%) but lower obesity rates (11.3% vs. 14.5%). Mean annual PM₂.₅ exposure was 44.1 ± 12.5 μg m^−3^. The 5th and 95th percentiles of the annual PM₂.₅ distribution were 25 and 62 μg m^−3^, respectively (Table 2), indicating that most participants were exposed to concentrations exceeding the Chinese Level 2 standard.

**Table 1:** Baseline characteristics of participants according to sleep-duration group.

Characteristic	Short sleep <6 h (*n* = 568)	≥6 h sleep (*n* = 1,514)	Total (*N* = 2082)	*p*-value
Age, years	71.6 ± 6.4	70.9 ± 6.4	71.1 ± 6.4	0.026
Female sex	285 (50.2)	621 (41.0)	906 (43.5)	<0.001
BMI, kg m^−2^	23.6 ± 3.8	23.9 ± 3.8	23.9 ± 3.8	0.109
Obesity (BMI ≥ 28)	73 (12.9)	203 (13.4)	276 (13.3)	0.794
Depressive symptoms	151 (26.6)	278 (18.4)	429 (20.6)	<0.001
Hypertension	347 (61.1)	924 (61.0)	1,271 (61.0)	1.000
Diabetes	145 (25.5)	263 (17.4)	408 (19.6)	<0.001
Ambient PM₂.₅, μg m^−3^	45.3 ± 12.2	42.7 ± 11.6	43.4 ± 11.8	<0.001
Smoking status – Never	328 (57.7)	987 (65.2)	1,315 (63.2)	0.007
Former	93 (16.4)	207 (13.7)	300 (14.4)	
Current	147 (25.9)	320 (21.1)	467 (22.4)	
Alcohol intake – Never	297 (52.3)	760 (50.2)	1,057 (50.8)	0.501
Occasional	177 (31.2)	513 (33.9)	690 (33.1)	
Regular	94 (16.5)	241 (15.9)	335 (16.1)	
Indoor solid-fuel use	105 (18.5)	294 (19.4)	399 (19.2)	0.675

**Table 2:** Adjusted odds ratios for short sleep across restricted-cubic-spline knots of PM₂.₅ exposure (Model 3).

Knot percentile (exposure)	Odds ratio (95% CI)
5th (25 μg m^−3^)	1.00
35th (38 μg m^−3^)	1.09 (0.97–1.23)
65th (48 μg m^−3^)	1.28 (1.07–1.53)
95th (62 μg m^−3^)	1.53 (1.18–1.98)

### 3.2 Prevalence of short sleep across PM₂.₅ categories

**Figure 1:**
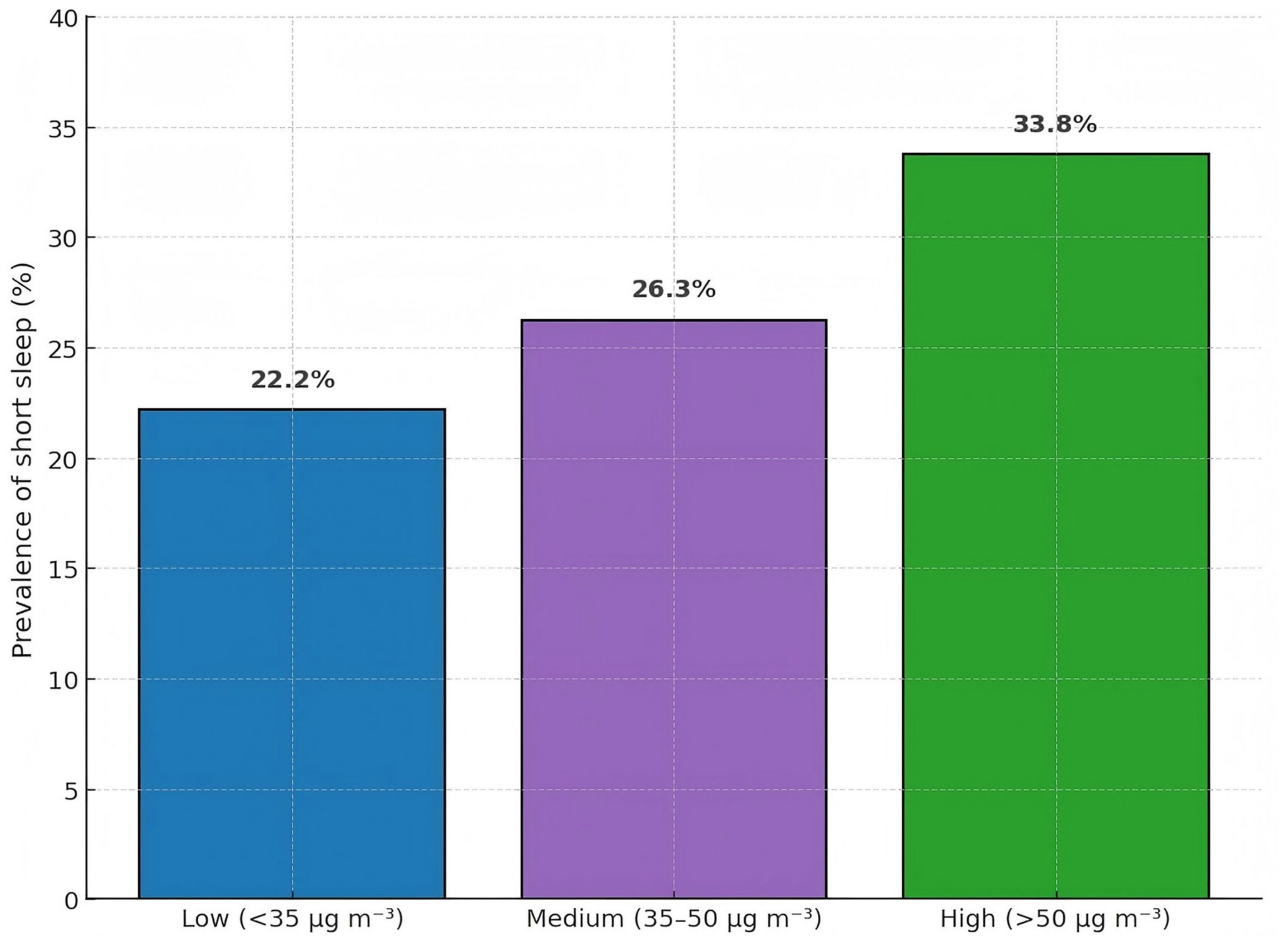
Prevalence of short sleep across low, medium, and high PM_2.5_ categories.

Short-sleep prevalence rose step-wise with increasing exposure (Table 3 and Figure 1): 21.8% in the low-exposure group (<35 μg m^−3^; 138/632), 25.5% in the medium group (35–50 μg m^−3^; 209/819), and 30.9% in the high-exposure group (>50 μg m^−3^; 195/631) (*χ*^2^ = 16.9, *p* < 0.001).

**Table 3:** Prevalence of short sleep by annual PM₂.₅ exposure category.

PM₂.₅ category	Participants, *n*	Short sleep, *n* (%)
Low (<35 μg m^−3^)	632	140 (22.2)
Medium (35–50 μg m^−3^)	819	215 (26.3)
High (>50 μg m^−3^)	631	213 (33.8)

Chi-square test for trend across categories: *χ*^2^ = 16.8, *p* < 0.001.

### 3.3 Association between PM₂.₅ and short sleep

In mixed-effects logistic models with a random intercept for recruitment site (Table 4), each 10 μg m^−3^ increase in annual PM₂.₅ was associated with:

**Table 4:** Multivariable mixed-effects logistic regression of annual PM₂.₅ exposure and short sleep (<6 h night^−1^).

Exposure metric	Model 1 OR (95% CI)	Model 2 OR (95% CI)	Model 3 OR (95% CI)
Per 10 μg m^−3^ increment	1.12 (1.04–1.20)	1.12 (1.04–1.20)	1.12 (1.04–1.20)
Categorical PM₂.₅ (μg m^−3^)			
Low < 35 (ref)	1	1	1
Medium 35–50	1.18 (0.93–1.49)	1.08 (0.84–1.37)	1.03 (0.80–1.31)
High > 50	1.55 (1.22–1.98)	1.50 (1.18–1.92)	1.51 (1.18–1.94)
*P* for linear trend	0.001	0.004	0.005

Model 1: adjusted for age and sex.

Model 2: Model 1 + education, income, BMI, waist circumference, smoking, alcohol, tea intake, physical activity, hypertension, diabetes, depressive symptoms, chronic pain.

Model 3: Model 2 + mean ambient temperature, indoor solid-fuel use, and ambient noise annoyance.

Model 1 (age, sex): OR 1.12 (95% CI 1.04–1.20).

Model 2 (+ socioeconomic, lifestyle, cardiometabolic factors): OR 1.12 (1.04–1.20).

Model 3 (+ ambient temperature, noise annoyance, solid-fuel use): OR 1.12 (1.04–1.20).

Using categorical exposure, the fully-adjusted odds ratios were 1.03 (0.80–1.31) for the medium group and 1.51 (1.18–1.94) for the high group, relative to the low-exposure reference.

### 3.4 Dose–response relationship

**Figure 2:**
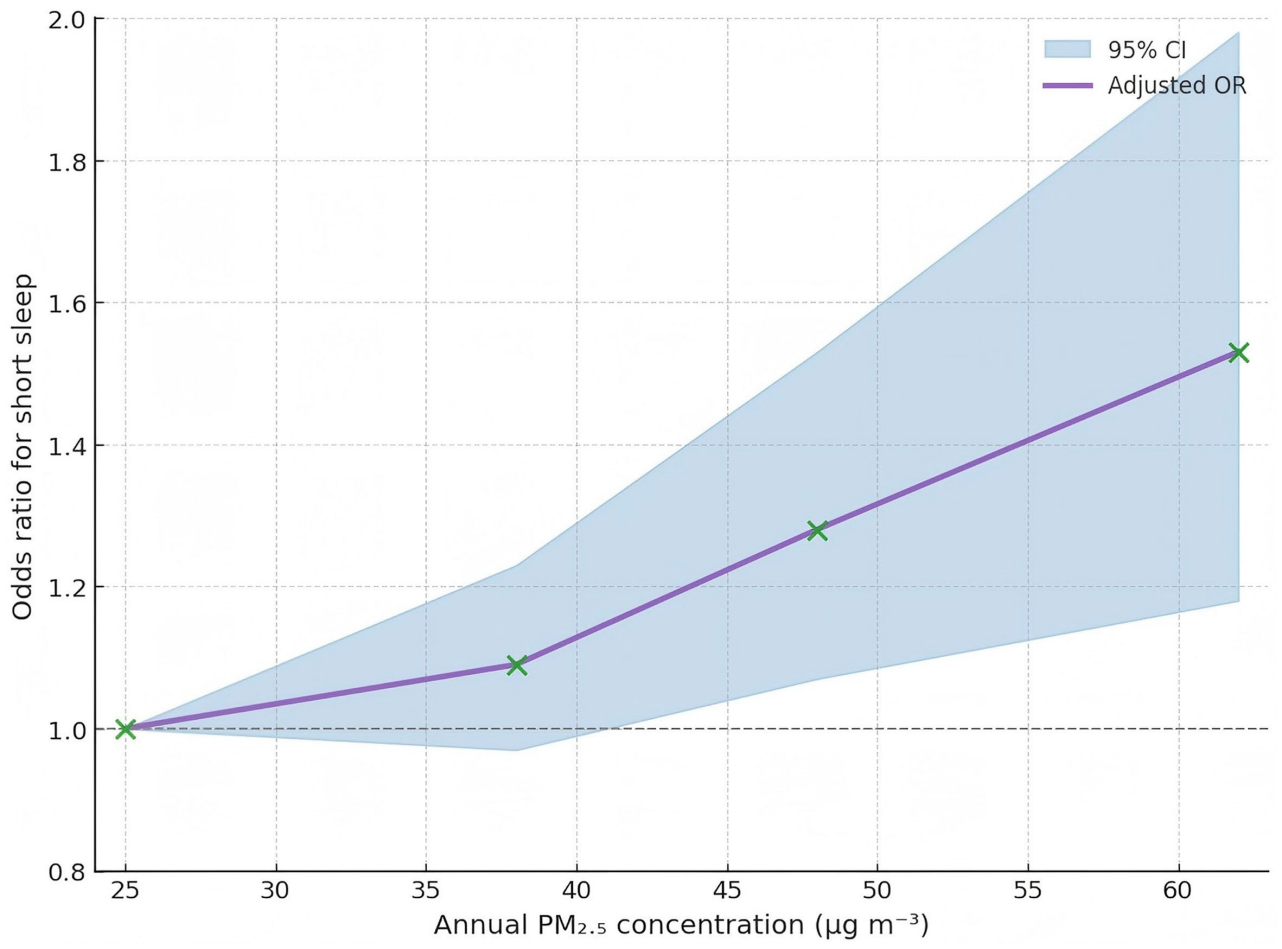
Dose–response curve for PM_2.5_ and odds of short sleep.

Restricted cubic-spline modeling demonstrated a monotonic, approximately log-linear rise in the odds of short sleep across the observed PM₂.₅ range without clear threshold effects (Figure 2). Likelihood-ratio tests comparing spline and linear specifications did not suggest marked departures from linearity, and knot-specific estimates are summarized in Table 2.

### 3.5 Effect modification

Formal interaction testing (Table 5) provided no evidence that the PM₂.₅–sleep association differed by sex (P_interaction = 0.62) or general obesity status (P_interaction = 0.48). A marginal interaction was seen with depressive symptoms (P_interaction = 0.09): the OR per 10 μg m^−3^ was 1.23 (1.03–1.45) among participants with depressive symptoms versus 1.09 (1.01–1.19) in those without, although the directionality was consistent across strata.

**Table 5:** Effect-modification analyses of the PM₂.₅–short-sleep association (Model 3).

Effect modifier (stratum)	Participants, *n*	Short-sleep cases, *n*	OR per 10 μg m^−3^ (95% CI)	*P*-interaction
Sex				0.62
Men	1,176	283	1.11 (1.02–1.21)	
Women	906	285	1.13 (1.02–1.26)	
General obesity (BMI ≥ 28 kg m^−2^)				0.48
No	1806	504	1.11 (1.03–1.20)	
Yes	276	64	1.15 (0.98–1.35)	
Depressive symptoms				0.09
Absent	1,653	417	1.09 (1.01–1.19)	
Present	429	151	1.23 (1.03–1.45)	

### 3.6 Sensitivity analyses

Results proved robust in multiple scenarios (Table 6). The OR per 10 μg m^−3^ was 1.05 (0.95–1.16) when short sleep was re-defined as <5 h, 1.10 (1.02–1.19) after excluding 5% of participants who self-reported physician-diagnosed insomnia, 1.15 (0.99–1.33) in two-pollutant models controlling for nitrogen dioxide, and 1.13 (0.93–1.39) when actigraphy-derived sleep duration was used in the 280-person sub-sample.

**Table 6:** Sensitivity analyses for the association between PM₂.₅ and short sleep (<6 h night^−1^).

Analysis scenario	Sample size, *n*	OR per 10 μg m^−3^ (95% CI)	*P*
Primary analysis (Model 3)	2082	1.12 (1.04–1.20)	<0.001
Short sleep defined as <5 h	2082	1.05 (0.95–1.16)	0.3
Excluding physician-diagnosed insomnia	1978	1.10 (1.02–1.19)	0.009
Two-pollutant model (PM₂.₅ + NO₂)	2082	1.15 (0.99–1.33)	0.067
Actigraphy-derived sleep (subset)	280	1.13 (0.93–1.39)	0.22

### 3.7 Population-attributable fraction and model performance

Assuming a counterfactual of PM₂.₅ ≤ 35 μg m^−3^, 24.0% of short-sleep cases were attributable to excess PM₂.₅ exposure (Table 7). Adding PM₂.₅ to a base model containing age, sex and recruitment site improved discrimination from a C-index of 0.55 to 0.57 (*Δ* 0.02, 95% CI 0.01–0.04; likelihood-ratio *p* = 0.012) and yielded a continuous net re-classification improvement of 0.07 (Table 8). Calibration plots showed good agreement between predicted and observed probabilities (Hosmer–Lemeshow *p* = 0.46).

**Table 7:** Population-attributable fraction (PAF) of short sleep attributable to annual PM₂.₅ > 35 μg m^−3^.

Exposure scenario	Short-sleep cases (*n*)	Estimated cases if PM₂.₅ ≤ 35 μg m^−3^ (*n*)	Excess cases (*n*)	PAF % (95% CI)
Observed population (*N* = 2082)	568	432	136	24.0 (20.5–27.5)
Medium group (35–50 μg m^−3^)	215	181	34	6
High group (>50 μg m^−3^)	213	109	104	18

**Table 8:** Incremental predictive performance gained by adding annual PM₂.₅ to a baseline clinical model for short sleep (*N* = 2082).

Metric	Baseline model	Baseline + PM₂.₅	Increment (95% CI)
C-index/AUROC	0.55	0.569	0.019 (0.012–0.026)
Continuous net re-classification improvement (NRI)	–	0.067	0.067 (0.015–0.119)
Integrated discrimination improvement (IDI)	–	0.006	0.006 (0.002–0.010)
Brier score	0.181	0.178	−0.003
Hosmer–Lemeshow *χ*^2^ (df = 8)	11.2	9.4	–

## 4 Discussion

This cross-sectional survey of more than 2,000 community-dwelling Chinese older adults provides novel population-based evidence that chronic exposure to fine-particulate air pollution associates with materially higher odds of habitual short-sleep duration, even after extensive adjustment for sociodemographic, lifestyle, cardiometabolic and environmental factors. A monotonic dose–response pattern was apparent: compared with residents breathing < 35 μg m^−3^ of annual PM₂.₅, those exposed to concentrations > 50 μg m^−3^ exhibited a 51% elevation in fully-adjusted odds of sleeping fewer than 6 h per night, while each 10 μg m^−3^ increment corresponded to a 12% rise in risk. Spline modeling further demonstrated a near-log-linear trajectory without threshold effects across the observed exposure range, and population-attributable calculations suggested that almost one quarter of short-sleep cases in this cohort could be prevented under compliance with China’s Level-2 air-quality standard. Collectively, these findings extend a growing but still limited literature by documenting a discernible sleep burden of ambient particulate pollution in a large, well-characterized sample of older adults, a demographic in whom restorative sleep is already compromised by age-related physiologic change (26–28). From a broader behavioral and social perspective, these findings fit within an environment–behavior–sleep framework in which chronic exposure to polluted, noisy neighborhoods can heighten physiological arousal, restrict outdoor and social activities, and erode the regular routines that support adequate sleep duration in late life.

Our results align with, yet also amplify, prior epidemiologic reports linking traffic-related or urban background PM₂.₅ to reduced actigraphic sleep efficiency, longer sleep-onset latency, and greater insomnia symptomatology in middle-aged populations (29–31). Recent cohort studies from the United States and Europe observed 7–10% increases in incident short sleep per interquartile rise in 24-h PM₂.₅, albeit in markedly cleaner settings (median 10–15 μg m^−3^) and younger adults (11, 28, 32–35). By contrast, the present investigation captured exposures that frequently exceeded 50 μg m^−3^. The magnitude of association we observed therefore corroborates a biologically plausible gradient while underscoring the disproportionate sleep health burden borne by residents of heavily polluted areas. In absolute terms, the mean annual PM₂.₅ concentration in our cohort (44.1 μg m^−3^) was roughly three times higher than typical levels reported for recent North American and European cohorts [approximately 10–15 μg m^−3^ (11, 28, 32–35)], underscoring the particularly heavy pollution context in which these older adults live.

Several interrelated pathophysiologic pathways may underpin the observed associations. First, inhaled ultrafine particulates activate pulmonary macrophages and trigger systemic release of pro-inflammatory cytokines—interleukin-6, tumor-necrosis-factor-α, and C-reactive-protein—that can penetrate the blood–brain barrier and perturb sleep-regulatory nuclei within the hypothalamus (36–39). Second, oxidative stress elicited by particulate transition metals and organic compounds dampens nocturnal melatonin synthesis and disrupts circadian gene expression, thereby shortening total sleep time (40–42). Third, autonomic imbalance characterized by sympathetic over-activity and reduced heart-rate-variability, well documented following acute PM₂.₅ spikes, may delay sleep initiation and fragment maintenance sleep through nocturnal arousals (40, 43–45). Collectively, these mechanisms converge on the clinical phenotype of short sleep duration and could operate synergistically with age-related vulnerabilities such as diminished anti-oxidant defenses and heightened neuro-inflammatory tone.

Effect-modification analyses suggested broadly consistent associations across sex and obesity strata, whereas a borderline-significant amplification was seen among participants reporting depressive symptoms. Neuro-inflammation and monoaminergic dysregulation represent shared biological substrates of air-pollution toxicity and late-life depression (46–51); hence, comorbid affective burden may heighten susceptibility to pollution-induced sleep impairment. Although the interaction did not achieve conventional statistical significance, the direction and magnitude of effect warrant replication in longitudinal studies with formal mediation analysis to disentangle the temporal sequence between pollution, affective symptoms and sleep disturbance. Nevertheless, we did not perform formal mediation analyses to quantify the extent to which depressive symptoms or other intermediate phenotypes might lie on the pathway between chronic PM₂.₅ exposure and short sleep duration; longitudinal data with repeated measurements will be needed to disentangle these interrelations more rigorously.

Key strengths bolster the credibility of our findings. The study leveraged a high-resolution satellite–ground-fusion exposure model (mean cross-validated *R*^2^ = 0.83), thereby minimizing spatial misclassification relative to conventional city-wide averages. Rigorous mixed-effects regression accounted for recruitment-site clustering, and a comprehensive battery of socioeconomic, behavioral, cardiometabolic and environmental covariates—captured through standardized protocols—reduced confounding. Construct validity of the sleep questionnaire was supported by moderate correlation with wrist actigraphy, and multiple sensitivity analyses, including alternative sleep thresholds, two-pollutant control for NO₂, and actigraphy-derived outcomes, yielded materially similar estimates, underscoring robustness. Notwithstanding these strengths, several limitations merit consideration. The cross-sectional design precludes causal inference; while short sleep is unlikely to raise ambient PM₂.₅ levels, residual reverse causation via time-activity patterns cannot be dismissed. Sleep duration was self-reported for the majority and may have incurred non-differential misclassification; nonetheless, such error would bias associations toward the null. Annual average PM₂.₅ may obscure critical exposure windows or indoor infiltration dynamics—particularly pertinent because we lacked direct measures of bedroom air quality, ventilation practices or time spent outdoors. Despite adjusting for solid-fuel use and ambient temperature, unmeasured co-pollutants (e.g., ultrafine particles, black carbon) or psychosocial stressors might confound associations. Finally, participants were older Han Chinese living in two south-western cities; generalisability to younger, rural, or ethnically diverse populations requires caution. In addition, we assessed only one dimension of sleep health—nightly sleep duration—rather than multidimensional constructs such as sleep efficiency, timing or fragmentation, so our outcome should be interpreted strictly as short sleep duration rather than global sleep quality. Although the exposure model generated daily PM₂.₅ estimates, our primary analyses used a single annual average and therefore did not capture potential short-term (e.g., day-to-day) effects on sleep, nor did we have harmonized high-resolution data for other major pollutants (such as O₃, PM₁₀, SO₂ and CO) beyond NO₂. True personal exposure is also shaped by behaviors—including time spent outdoors, window opening and the use of air conditioning or air cleaners—that we were unable to characterize in detail. Residential histories were not collected, so chronic exposure may be misclassified for participants who had recently moved. Recruitment through a hospital-based outreach program may have preferentially attracted individuals who are more health-conscious or who have existing health conditions, which could limit the generalisability of our findings. Finally, we restricted formal effect-modification analyses to a small set of *a priori* factors (sex, obesity and depressive symptoms) to avoid excessive multiple testing, and we did not present detailed spatial or temporal maps of PM₂.₅ exposure, which might have further illustrated exposure contrasts across the study region.

Higher chronic exposure to fine-particulate air pollution is associated with appreciably greater prevalence of short sleep among community-dwelling Chinese elders, following a clear dose–response gradient that persists across numerous sensitivity analyses. From a public-health perspective, our data imply that stringent enforcement of national PM₂.₅ standards could confer substantive sleep-health benefits alongside cardiopulmonary gains. Clinicians and policymakers should therefore consider ambient air quality when evaluating sleep complaints in older adults and integrate pollution mitigation—be it regional emission control or household air-cleaning interventions—into multifaceted strategies aimed at preserving healthy sleep across the aging trajectory. Prospective longitudinal cohorts and experimental paradigms are now required to delineate critical exposure windows, clarify mechanistic pathways, and quantify the restorative impact of particulate-matter reduction on objectively measured sleep architecture and downstream geriatric outcomes.

## 5 Conclusion

The current study delineated the associations between chronic ambient fine-particulate matter (PM₂.₅) exposure and habitual short sleep duration in 2082 community-dwelling Chinese older adults. Each 10 μg m^−3^ increment in annual PM₂.₅ corresponded to a 12% elevation in the odds of sleeping fewer than 6 h per night, while exposure above 50 μg m^−3^ conferred a 51% excess risk relative to concentrations below 35 μg m^−3^, with dose–response linearity persisting across extensive sensitivity and interaction analyses. These findings cast ambient PM₂.₅ as a modifiable environmental determinant of restorative sleep and suggest that achieving national air-quality standards could avert nearly one quarter of cases of short sleep duration in this aging population. Clinicians, public-health practitioners and policymakers should therefore weave particulate-matter mitigation into multifaceted strategies aimed at safeguarding sleep health alongside cardiopulmonary protection. Future longitudinal cohorts and targeted intervention trials that pair high-resolution exposure assessment with objective sleep metrics are now warranted to confirm causality, pinpoint vulnerable exposure windows, and quantify the restorative impact of PM₂.₅ reduction on geriatric sleep architecture and downstream health trajectories.

## Data Availability

The original contributions presented in the study are included in the article/supplementary material, further inquiries can be directed to the corresponding author.
